# Cartilage tissue turnover increases with high- compared to low-intensity resistance training in patients with knee OA

**DOI:** 10.1186/s13075-023-03000-2

**Published:** 2023-02-10

**Authors:** Christian S. Thudium, Amalie Engstrøm, Anne-Christine Bay-Jensen, Peder Frederiksen, Nuria Jansen, Arjan De Zwart, Marike van der Leeden, Joost Dekker, Willem Lems, Leo Roorda, Willem Evert van Spil, Martin Van der Esch

**Affiliations:** 1 Immunoscience, Nordic Bioscience, Herlev, Denmark; 2 Faculty of Health and Medical Sciences, University of Copenhagen, Copenhagen, Denmark; 3 Reade Centre for Rehabilitation and Rheumatology, Amsterdam, the Netherlands; 4 Department of Rehabilitation Medicine, Amsterdam Institute of Public Health, Amsterdam UMC, Vrije Universiteit Amsterdam, de Boelelaan, 1117 Amsterdam, the Netherlands; 5 Department of Rheumatology, Amsterdam UMC, Vrije Universiteit Amsterdam, Amsterdam, the Netherlands; 6 Dijklander Hospital, Hoorn, the Netherlands; 7 University Medical Center Utrecht, Utrecht, the Netherlands; 8 Centre of Expertise Urban Vitality, Faculty of Health, Amsterdam University of Applied Sciences, Amsterdam, the Netherlands

## Abstract

**Objectives:**

To investigate cartilage tissue turnover in response to a supervised 12-week exercise-related joint loading training program followed by a 6-month period of unsupervised training in patients with knee osteoarthritis (OA). To study the difference in cartilage tissue turnover between high- and low-resistance training.

**Method:**

Patients with knee OA were randomized into either high-intensity or low-intensity resistance supervised training (two sessions per week) for 3 months and unsupervised training for 6 months. Blood samples were collected before and after the supervised training period and after the follow-up period. Biomarkers huARGS, C2M, and PRO-C2, quantifying cartilage tissue turnover, were measured by ELISA. Changes in biomarker levels over time within and between groups were analyzed using linear mixed models with baseline values as covariates.

**Results:**

huARGS and C2M levels increased after training and at follow-up in both low- and high-intensity exercise groups. No changes were found in PRO-C2. The huARGS level in the high-intensity resistance training group increased significantly compared to the low-intensity resistance training group after resistance training (*p* = 0.029) and at follow-up (*p* = 0.003).

**Conclusion:**

Cartilage tissue turnover and cartilage degradation appear to increase in response to a 3-month exercise-related joint loading training program and at 6-month follow-up, with no evident difference in type II collagen formation. Aggrecan remodeling increased more with high-intensity resistance training than with low-intensity exercise.

These exploratory biomarker results, indicating more cartilage degeneration in the high-intensity group, in combination with no clinical outcome differences of the VIDEX study, may argue against high-intensity training.

## Introduction

A major step forward in designing effective exercise therapy interventions for knee OA patients is to better understand the mechanisms why exercise therapy helps to alleviate OA symptoms, including a better understanding of the direct effect exercise therapy has on the cartilage turnover of the knee joint [1].

A direct effect of exercise-related joint loading is believed to have a significant effect on cartilage turnover [2]. This belief is based on studies examining cartilage turnover, but with frequently contradictory results. High joint loading by sport showed an increased total cartilage surface area and increased thickness of patellar cartilage [3, 4]. However, knee joint unloading and inactivity showed cartilage thinning [5, 6]. In persons at risk of developing OA, exercise-related joint loading was associated with an increased risk of patellar cartilage volume loss and an increased cartilage glycosaminoglycan (GAG) content [7, 8]. To gain more insight into the effects of high exercise-related joint loading, a previously performed randomized clinical trial (RCT), the VIDEX trial, provided an opportunity to study the effects of cartilage turnover in response to exercise-induced joint loading, by studying cartilage biomarkers [9]. Biomarkers related to degradation and synthesis of cartilage extracellular matrix proteins have allowed sensitive quantification of metabolic changes in the cartilage tissue in response to interventions [10]. Because of the conflicting results and because it was not the primary aim of the VIDEX trial, no predefined hypothesis was formulated.

Aggrecan and type II collagen are the two most abundant extracellular matrix proteins of the cartilage. In healthy subjects, there is a delicate balance between the remodeling of these components ensuring cartilage tissue homeostasis. In OA, a hallmark of the disease is the degradation of cartilage due to increased proteolytic activity. This activity results in the release of specific extracellular degradation fragments. In the VIDEX study, the a disintegrin and metalloproteinase with a thrombospondin type 1 motif (ADAMTS) generated aggrecan marker huARGS was used to quantify aggrecan degradation. Formation and degradation of type II collagen were assessed with the biomarkers PRO-C2 and C2M, respectively. These three biomarkers have previously been measured in serum samples from clinical OA trials and have shown to reflect changes in cartilage tissue turnover [11–16]. In the VIDEX study, cartilage-related biomarkers were quantified to examine the impact of high-intensity (HI) versus low-intensity (LI) resistance training (RT). It can be speculated that the HI resistance training would result in higher exercise-related knee joint loading and therefore impact cartilage turnover more than LI resistance training. Therefore, changes in biomarkers resulting from both RT interventions, high versus low, were assessed.

The objective of this study was to investigate cartilage tissue turnover in patients with knee OA in response to a supervised 12-week exercise-related joint loading training program followed by a 6-month period of unsupervised gym training. A second objective was to study the difference in cartilage tissue turnover between HI and LI resistance training.

## Methods

### Study design and patients

**Fig. 1:**
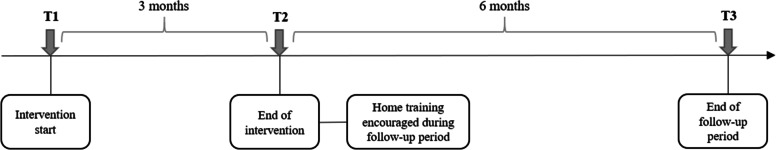
Overview of the study design

This study represents a secondary analysis of the VIDEX trial (registration number: NL47786.048.14, EudraCT Number: 2014-000047-33) [9]. The VIDEX trial was a single-blinded randomized controlled trial including 177 patients of older age (between 55 and 80 years) diagnosed with knee OA according to ACR criteria [17]. In total, there were 12 exclusion criteria including other forms of arthritis than OA, absolute contra-indications for exercise due to uncontrolled high blood pressure or other co-morbidities, performed or scheduled total knee arthroplasty and/or strength training in the past 3 months, and psychoneuroticism [9]. Patients were allowed to take pain medication before and during the study. Patients were randomized into two groups: HI or LI RT. In both the HI and LI RT groups, patients trained in 3 sessions per week with a focus on upper leg muscle strength. In the HI RT group, patients performed the exercises at 70–80% of their individual 1 repetition maximum (1RM), whereas patients in the LI RT group were instructed to perform at 40–50% of their individual 1RM. For 12 weeks, patients performed a supervised resistance training intervention, whereafter they were encouraged to continue the training program independently during the following 6 months. Serum samples were collected before the training start (PRE-XT), after the 12-week training intervention (POST-XT), and after the 6-month follow-up period (FU) as depicted in Fig. 1. Samples were stored at −80 °C until biomarker assessment.

**Fig. 2:**
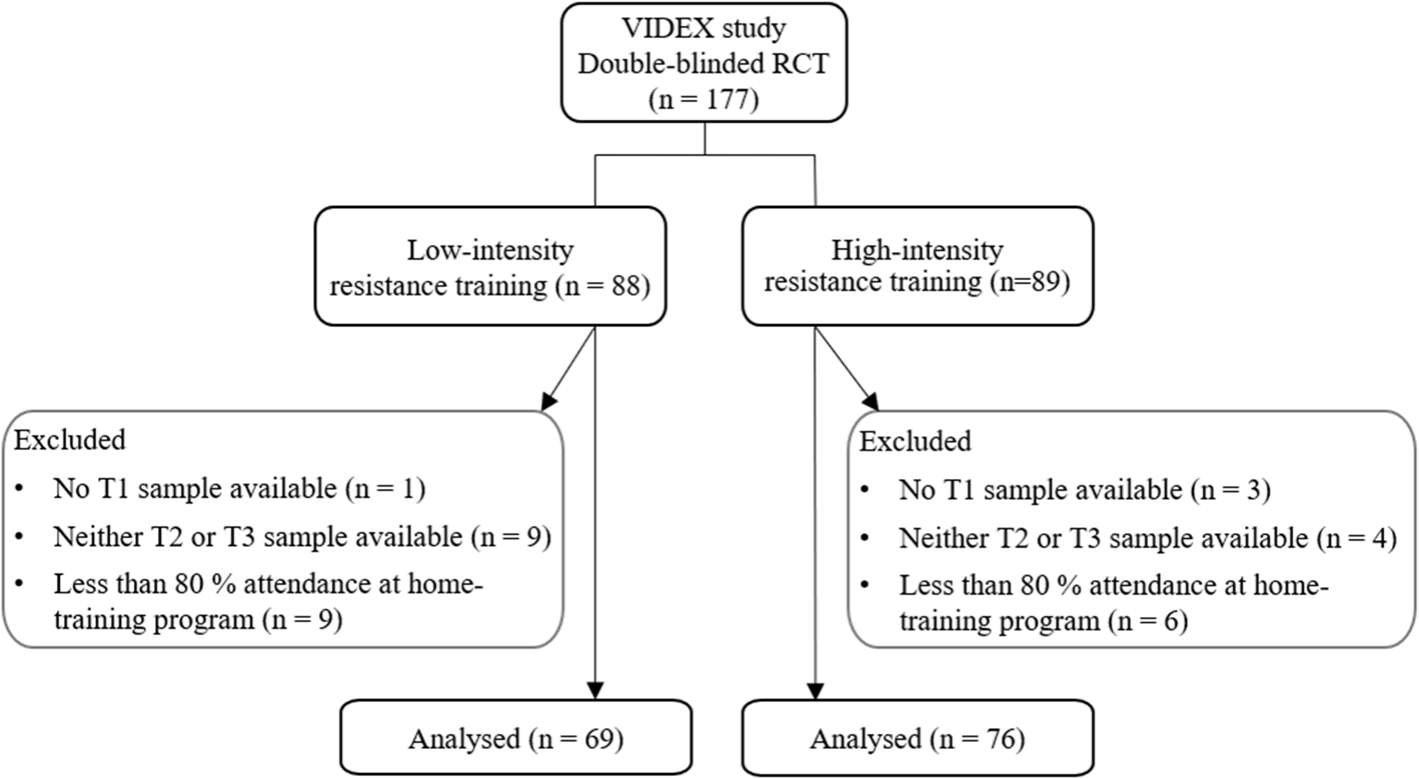
CONSORT diagram of the study patients

Of the 177 patients included in the study, PRE-XT samples were not available for 4 persons. In addition, samples from a total of 13 patients were lost to follow-up and had neither POST-XT nor FU samples available. Furthermore, patients who failed to complete a minimum of 80% of the scheduled home training program during the 6 months between POST-XT and FU were excluded from the analysis. Thus, 145 patients were analyzed, hereunder 76 patients in the HI RT group and 69 patients in the LI RT group. The CONSORT diagram is shown in Fig. 2.

### Biomarkers

Three cartilage turnover biomarkers were measured in the serum: huARGS quantifies ADAMTS-mediated aggrecan degradation by targeting a neoepitope of the aggrecan G2 domain. C2M measures a matrix-metallo-proteinase (MMP) generated neoepitope of type II collagen indicative of collagen degradation. PRO-C2 measures type II collagen formation via targeting the type IIB N-terminal pro-peptide region. Human ARGS (huARGS) was assessed by a chemiluminescence sandwich ELISA [18], C2M was measured with a competitive ELISA [19], and PRO-C2 was measured using a competitive electrochemiluminescence assay [20] (Nordic Bioscience, Herlev, Denmark). The measurements were performed in duplicate and blinded to the assigned HI or LO RT groups. Sample duplicates with a coefficient of variance (CV) above 15% were rerun. For each plate, three internal and two kit controls were measured to observe the intra- and inter-assay CV. Intra- and inter-CV% were huARGS: ≤2.1% and ≤ 4.8–9.6%, C2M: ≤ 4.8% and ≤19.6%, and PRO-C2: ≤ 5.4% and ≤ 13.6%.

### Statistical analysis

HI and LI RT groups were compared using *t*-tests with Bonferroni adjustment or chi-square test as appropriate. For all analyses, biomarker data were log-transformed to obtain normal distributions. Differences in biomarker levels within and between HI and LI RT groups at POST-XT and FU were assessed using a linear mixed model with interaction between the two fixed effects intensity and time. Patient ID was included as a random effect and PRE-XT biomarker level as a covariate. Multiple pairwise comparisons were adjusted with Tukey’s test. Statistical tests were performed in R version 4.1.0 (R Foundation for Statistical Computing, Vienna, Austria) and RStudio version 1.4.1106 (RStudio, PBC, Boston, MA). The results were plotted in GraphPad Prism version 9.1.2 (GraphPad Software, San Diego, CA).

## Results

### Patients

The baseline characteristics of patients are summarized in Table 1. Approximately 70% of the patients had bilateral knee OA. Overall, there were no significant differences between the two groups. There were more patients with low Kellgren and Lawrence (KL) scores in the HI RT and LI RT groups (39 and 30% respectively, with KL grade 1). There were no differences in biomarker levels at baseline between the two groups.

**Table 1:** Patient demographics at PRE-XT

	High-intensity resistance training		Low-intensity resistance training	
	N	Mean ± SD or n (%)	N	Mean ± SD or n (%)
Age(yrs)	76	67.4 ± 5.5	69	68.4 ± 5.9
BMI(kg/m2)	76	28.4 ± 4.3	69	27.9 ± 4.7
Female	76	48 (63.2 %)	69	40 (58.0 %)
Bilateral OA	76	73 (96.1 %)	69	66 (95.7 %)
WOMAC pain	76	6.4 ± 3.5	66	6.3 ± 3.8
WOMAC physical function	76	20.8 ± 12.2	67	20.8 ± 13.0
WOMAC stiffness	76	3.4 ± 1.8	67	3.2 ± 2.0
KL grade 1	76	30 (39.5 %)	69	21 (30.4 %)
KL grade 2	76	30 (39.5 %)	69	18 (26.1 %)
KL grade 3	76	8 (10.5 %)	69	15 (21.7 %)
KL grade 4	76	7 (9.2 %)	69	15 (21.7 %)
huARGS	76	184.4 (60.0)	69	178.6 (67.5)
C2M	76	0.20 (0.07)	69	0.21 (0.07)
PRO-C2	76	24.2 (8.5)	69	22.8 (5.1)

### The effect of resistance training on biomarker levels

The changes in huARGS and C2M levels from PRE-XT to POST-XT and from PRE-XT to FU were used as outcomes in the applied mixed model analysis. Both huARGS and C2M levels increased from PRE-XT to FU. Within the HI RT group, huARGS levels increased POST-XT by 25% (*p* < 0.0001) and by 50% (*p* < 0.0001) at FU (Table 2). The huARGS levels increased by 9% (*p* = 0.045) at POST-XT and 24% (*p* < 0.0001) at FU in the LI RT group. In the HI RT group, C2M levels were elevated at POST-XT by 27% (*p* < 0.0001) and by 42% (*p* < 0.0001) at FU. The C2M levels increased by 17% (*p* < 0.0001) at POST-XT and 54% (*p* < 0.0001) at FU in the LI RT group. For PRO-C2, no change in serum levels was observed from PRE-XT to FU within both groups.

**Table 2:** Effect of RT on biomarker levels from PRE-XT to POST-XT and FU. Serum samples were collected before RT start (T1), after 3-month supervised RT (T2), and after 6-month home training (T3). The biomarker data were log-transformed, and the difference in biomarker levels from PRE-XT to POST-XT and from PRE-XT to FU was calculated and used as an outcome as a model was fitted to the data using linear mixed models. The difference in biomarker levels was used to calculate p values using probability cumulative analysis on Student’s t-distribution

	T2			T3		
	Percent change from T1			Percent change from T1		
	Mean	95% CI	*p*-value	Mean	95% CI	*p*-value
**High-intensity resistance training**						
Log huARGS	25.0	15.73, 35.11	**<0.0001**	50.2	15.73, 62.28	**<0.0001**
Log C2M	27.9	18.67, 38.05	**<0.0001**	42.0	31.24, 53.64	**<0.0001**
Log PRO-C2	-3.9	-9.56, 2.16	0.203	5.7	-0.83, 12.60	0.883
**Low-intensity resistance training**						
Log huARGS	8.7	0.186, 17.86	**0.045**	23.9	13.80, 34.90	**<0.0001**
Log C2M	17.8	8.80, 27.51	**0.0001**	54.1	41.77, 67.39	**<0.0001**
Log PRO-C2	-6.0	-11.80, 0.24	0.059	2.6	-8.88, 4.20	0.447

### The effect of training intensity on biomarker levels

**Fig. 3:**
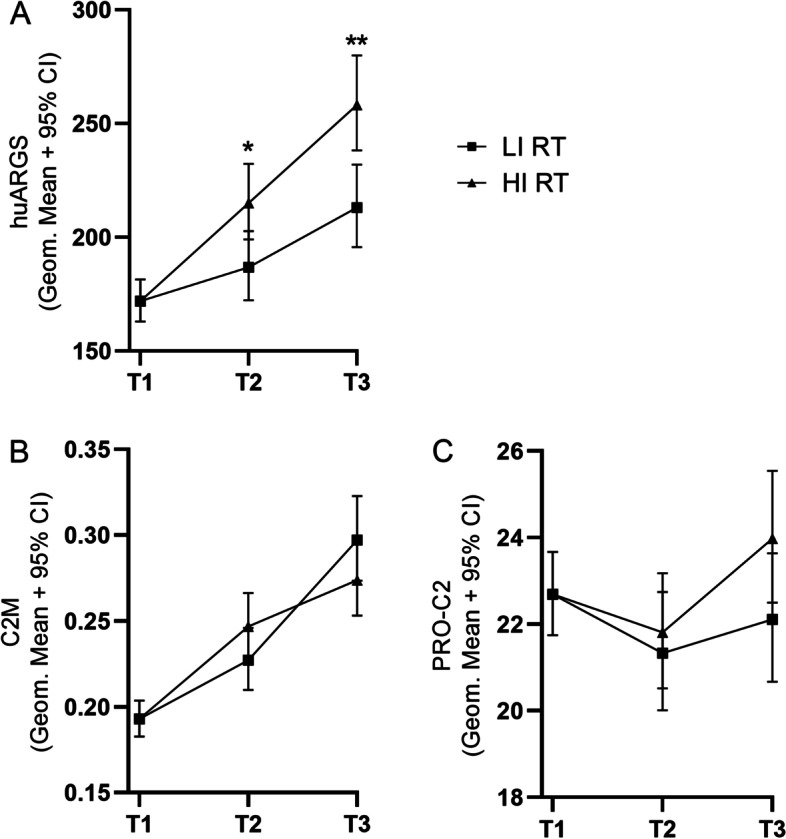
Effect of high-intensity (HI) and low-intensity (LI) resistance training (RT) on cartilage-related biomarkers huARGS, C2M and PROC2. Serum samples were collected before RT start (PRE-XT), after 3-month supervised RT (POST-XT), and after 6-month home training (FU). The biomarker data was log-transformed, and using a linear mixed model, a model was fitted. PRE-XT values are used as covariates; hence, the mean of PRE-XT biomarker values across the two groups is plotted here. Log-transformed data are presented on a linear *y*-axis as mean and 95% confidence intervals (CI). Group mean differences were assessed with linear mixed models with Tukey adjustment and the significance levels are presented with asterisks: *p < 0.05, **p < 0.01 which *p* value: versus baseline, or high versus low intensity

Only huARGS serum levels significantly differed at POST-XT and FU between the HI and LI RT groups (Fig. 3A); the HI RT group showed an increase in huARGS of 15% (*p* = 0.014) at POST-XT and an increase of 21% (*p* = 0.0014) at FU, compared to the LI RT group. Neither C2M nor PRO-C2 levels differed between the two training groups (Fig. 3B, C).

## Discussion

The present study investigated cartilage-related biomarkers in serum from knee OA patients participating in either HI RT or LI RT. We assessed changes in aggrecan and type II collagen biomarker levels during training intensity programs. Our main findings were that both huARGS and C2M biomarker levels in serum increased with resistance training within the HI as well as the LI exercise groups and that huARGS levels increased more after 3 months of supervised training and after 6 months of home training in the HI RT group compared to the LI RT group. These data suggest that RT induces alterations in the cartilage tissue turnover in knee OA patients. Interestingly, the increase in huARGS and C2M levels continued from POST-XT to FU despite the transition from supervised training to unsupervised training.

The cartilage turnover and degradation seem to be increased during high-intensity training, not only during the training, measurement at 3 months, but also after additional 3 months of follow-ups, which means relatively long-term elevated turnover.

The increased serum levels of huARGS and C2M could be a consequence of changes in cartilage tissue turnover from training. The increased huARGS levels observed in the HI RT group may represent an additional increase in tissue turnover. This increase in turnover was also found in C2M but not differently between the HI and LI RT groups. In the current study, aggrecan degradation or remodeling appeared to be more related to training intensity than the C2M biomarker. However, the increase in C2M levels in both HI and LI RT groups from PRE-XT to FU may suggest that RT induces a catabolic or remodeling effect in type II collagen in cartilage, but that response is more resistant to the magnitude of intensity. Evidence of an interplay between aggrecan remodeling and type II collagen formation was presented in an ex vivo study investigating the recombinant form of fibroblast growth factor 18 (FGF-18), Sprifermin, on human cartilage explants [21]. A peak in aggrecanase-mediated aggrecan degradation preceded PRO-C2 release. It has been suggested that aggrecan degradation and tissue remodeling allow space for chondrocyte proliferation which in turn enables cartilage formation to occur [22, 23]. The present study did not show a change in PRO-C2, but it is worth noting that structural changes in the cartilage matrix in response to stimulation, whether by exercise or by drug candidates, may occur in temporal phases [11, 21].

In the present study, we observed that the huARGS and C2M levels increased over time. Whether the change over time is a positive or a negative finding should be considered. It seems that high-intensity exercise has a stimulating effect on cartilage tissue turnover, but it is not clear whether these increased levels are part of a remodeling response or associated with net cartilage degradation. It is therefore difficult to draw conclusions, partly due to the design of this study in which a secondary analysis was performed in an RCT (the VIDEX study) [9]. This RCT did not include a control of healthy people. Therefore, it is not well known what effect HI RT has on cartilage turnover. The acute response of huARGS and C2M to exercise therapy has previously been investigated [14, 24]. Exercising a joint might affect the dynamics/kinetics of the biomarkers. If the cartilage load is higher, more biomarkers might be released from it. A study to support this showed an acute increase of blood biomarker levels right after some exercise. A similar mechanism might explain our findings [25]. In response to running or cycling, serum ARGS levels showed a slight elevation in the 3 h after exercise in both healthy controls and OA patients, while C2M showed a significant increase in the 3 h after cycling in OA patients [14, 24]. These changes in serum ARGS levels might be due to changes in tissue turnover but be just as well a consequence of changed biomarker dynamics (e.g., increased lymph flow from the joint because of exercise). Biochemical markers from joint tissue turnover and inflammatory markers have been shown to differ between OA patients and healthy subjects [26, 27]. Matrix degradation might be part of cartilage regeneration; in other words, both matrix degradation and synthesis might occur in the process of cartilage regeneration. We cannot fully support this alternative explanation for our finding with our own study results though, as PRO-C2 levels did not change. Our study did not have a control group of healthy subjects; thus, it is unclear which role OA plays in our observations. Previous findings showed similar changes in serum COMP levels in response to 30-min walking in both patients with knee OA and in healthy controls, suggesting that the acute response to moderate physical activity is similar in healthy subjects and OA patients [28]. It is clear that a long-term study is needed with contrast between the groups including a healthy, no joint-pain group.

It is worth considering that there might not be a linear relationship between training intensity and efficacy but perhaps that exercise in OA patients functions by a goldilocks principle of a “just right” amount. This range of beneficial exercise for OA patients may be defined by lower thresholds than for healthy subjects. Two longitudinal studies investigated the general recommendations of 10,000 steps/day or 150 min/week of moderate/vigorous activity and found that ≥10,000 steps/day was associated with degenerative effects and that the threshold for moderate/vigorous exercise needed for improving function was approximately half that recommended [29, 30]. Similarly, another longitudinal cohort study of subjects with or at risk for developing OA found that both sedentary lifestyle and high levels of physical activity were associated with degenerative cartilage changes [31]. Studies suggested that both too little and too much exercise are unhealthy for the cartilage tissue and importantly that this range of beneficial exercise may be lower in OA patients than in healthy subjects [29–31]. In the rheumatoid arthritis (RA) field, studies in RA patients subjected to increased intensity training find more structural joint deterioration in patients with severe RA at baseline compared to less severe. These findings may lead to speculation that more intense training in late-stage OA patients could prove detrimental [32]. The exact mechanisms behind this decreased threshold for exercise in persons with or at risk of OA remain to be elucidated; however, in vivo studies of mice which have undergone surgical destabilization of the medial meniscus (DMM) showed that joint loading and especially shear stress induced OA [33, 34]. This is perhaps enforced in subjects at risk of OA development, where obesity and previous joint trauma among others are factors that contribute to joint instability and malalignment which in turn may increase the shear stress applied to the knee joint. In the current study, previous knee surgery was not an exclusion criterion, but the number of patients having prior surgeries was balanced between the two groups. Similarly, the use of NSAIDS or injections was allowed, but was similar between groups. The use of NSAIDS could be important, as it might hypothetically affect levels of biomarkers. However, a recent study investigating the modulation of joint-related biomarkers in hand OA patients treated with prednisolone found no modulation [35].

The large number of patients and the longitudinal design of the VIDEX study provided a good opportunity to gain more insight into the change in biomarker levels as a result of knee joint loading through the exercises. However, there are several limitations of the study. The VIDEX randomized control trial did not include a control group which did not receive exercise therapy, and therefore, a comparison of the results to a group without exercise intervention was not possible. Furthermore, it will be important to better understand how biomarker response differs between OA patients and healthy controls subjected to a similar level of physical activity or exercise intervention. An important limitation is also that actual endpoints of structural damage are yet unknown. Biomarker levels are therefore difficult to interpret. It is also difficult to bridge the clinical relevance as it is not known whether increased degradation of aggrecan leads to net loss or whether it is a sign of remodeling and reinforcement of the cartilage. Therefore, future studies should also relate changes in biomarker levels with changes in the clinical parameters as a result of exercise, for example WOMAC functionality scores, knee pain severity, and performance tests.

In conclusion, cartilage tissue turnover appears to increase in response to an exercise-related joint loading training program with no evident difference in type II collagen turnover between high- and low-intensity exercises. Only aggrecan remodeling was found to increase with high-intensity resistance training. Given the absence of a favorable effect on clinical signs and symptoms in high versus low exercise-treated patients, these exploratory results indicate that the combination of clinical findings of the VIDEX study (no difference in clinical outcomes) and the findings on the biomarker analyses (more cartilage degeneration in the high-intensity group) argue against high-intensity training in knee OA patients.

## Data Availability

Not applicable.

## Abbreviations

OA: Osteoarthritis
GAG: Glucosaminoglycan
RCT: Randomized clinical trial
ADAMTS: A disintegrin and metalloproteinase with a thrombospondin type 1 motif
HI: High intensity
LI: Low intensity
RT: Resistance training
1RM: 1 repetition maximum
MMP: Matrix metalloproteinase
CV: Coefficient of variation
KL: Kellgren and Lawrence
FGF-18: Fibroblast growth factor 18
